# Supplementary material for: A Bronze-Tomato Enriched Diet Affects the Intestinal Microbiome under Homeostatic and Inflammatory Conditions
Source: Nutrients. 2018 Dec 2;10(12):1862. doi: 10.3390/nu10121862 (PMC6315348; doi:10.3390/nu10121862)
Supplement: Supplementary file 1 [file nutrients-10-01862-s001.pdf]

Supplementary Materials: A Bronze-Tomato Enriched Diet Affects the Intestinal Microbiome under Homeostatic and Inflammatory Conditions

Marina Liso, Stefania De Santis, Aurelia Scarano, Giulio Verna, Manuela Dicarlo, Vanessa Galleggiante, Pietro Campiglia, Mauro Mastronardi, Antonio Lippolis, Mirco Vacca, Anastasia Sobolewski, Grazia Serino, Eugenio Butelli, Maria De Angelis, Cathie Martin, Angelo Santino and Marcello Chieppa

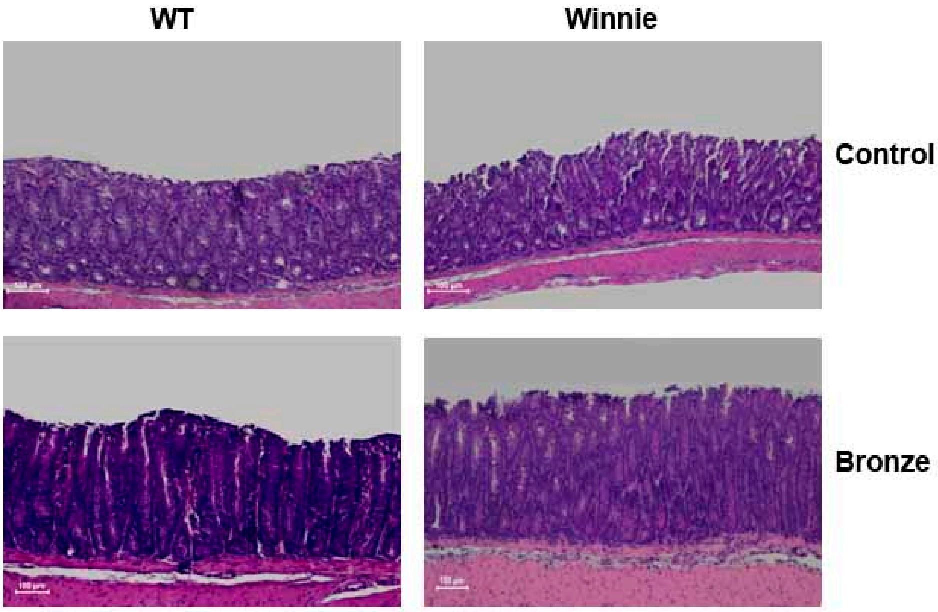

Figure S1. Histological characterization of the experimental groups. Representative images of dissected colons from WT and Winnie mice fed with Control or Bronze-enriched diets.

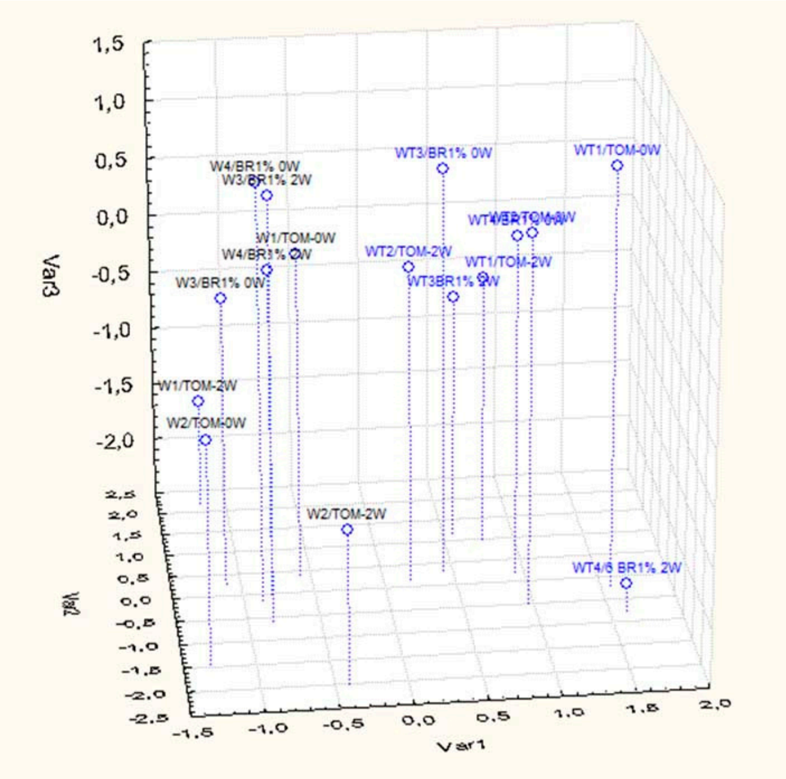

Figure S2. Principal component analysis (PCA) of total bacterial genera found in feces of mice. Score plot of the three principal components (PC) after principal component analysis (PCA) of total bacterial OTUs (16S rRNA), which were found in the fecal samples of WT (WT1-WT4 mice) and Winnie (W1-W4 mice) at T0 (0W) and after two weeks (2W) of Bronze (BR) or Control tomato (TOM) diets. Blue characters refer to OTUs of fecal samples of WT mice; black characters refer to OTUs of Winnie mouse fecal samples.

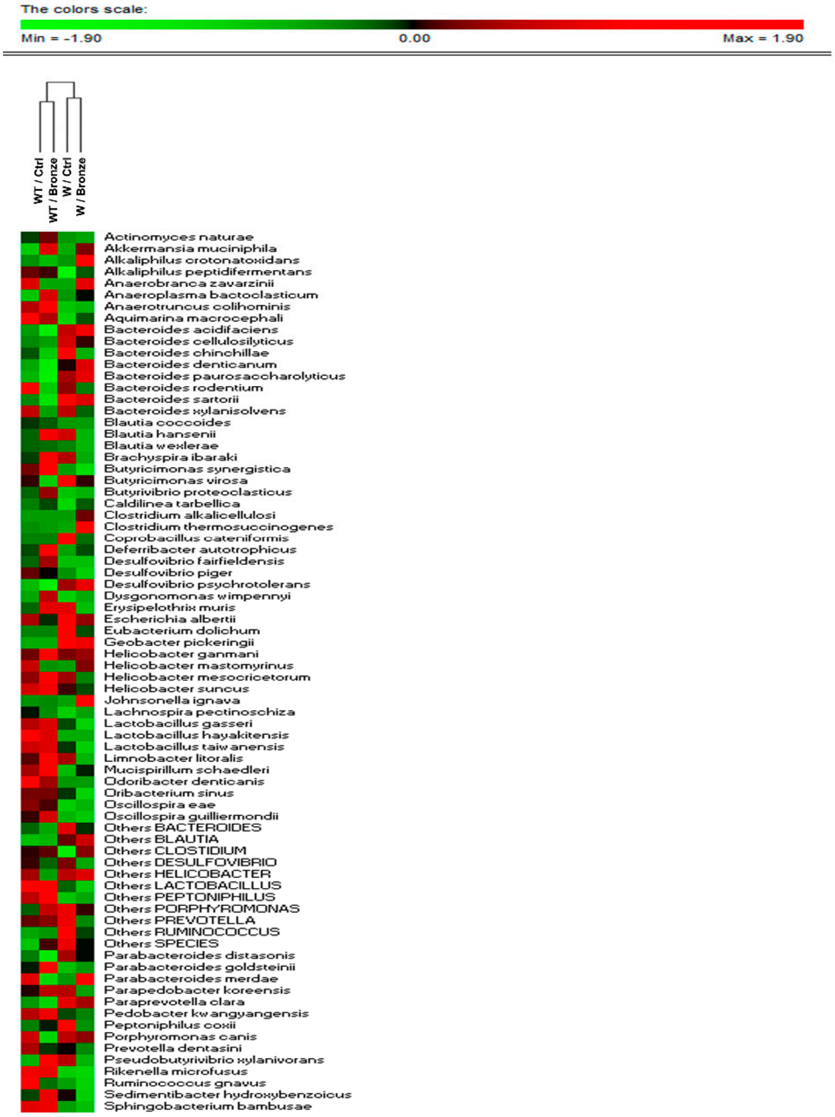

Figure S3. A Bronze-enriched diet affected the fecal bacterial species of WT and Winnie mice. Relative abundance (%) of total (16S rRNA) bacteria, which were differentially found at the species level in the fecal samples of WT and Winnie mice after two weeks of Bronze (Bronze) or Control tomato (Ctrl) supplemented diets.

Table S1. Number of identified species (OTUs), Shannon diversity index and Chao 1 mean of the microbiota of C57BL/6 (WT) and Winnie mice fed with Control tomato or Bronze tomato supplemented chow (Bronze). Values within a column with different superscript letters are significantly different ($P < 0.05$; FDR < 0.05).

Samples	Shannon Diversity Index	OTUs	Chao1
WT-Day 0 Control tomato	2.18b	253b	499a
Winnie-Day 0 Control tomato	2.41a	257b	507a
WT-2 Week Control tomato	2.30a	294a	534a
Winnie-2 Week Control tomato	2.39a	291ab	527a
WT-2 Week Bronze	2.20a	268b	503a
Winnie-2 Week Bronze	2.44a	267b	518a
